# Effect of Frying Process on Nutritional Property, Physicochemical Quality, and *in vitro* Digestibility of Commercial Instant Noodles

**DOI:** 10.3389/fnut.2022.823432

**Published:** 2022-02-17

**Authors:** Jin Wang, Ang Li, Jiaqiang Hu, Bowei Zhang, Jingmin Liu, Yan Zhang, Shuo Wang

**Affiliations:** Tianjin Key Laboratory of Food Science and Health, School of Medicine, Nankai University, Tianjin, China

**Keywords:** frying process, instant noodle, nutritional property, physicochemical quality, *in vitro* digestibility

## Abstract

The effects of frying process on the nutritional property, physicochemical quality, and *in vitro* digestibility of instant noodle products are investigated in this study. Scanning electron microscope (SEM) and Fourier transform infrared spectrometer (FT-IR) were also used to explore the changes in the microstructure and protein transformation. Noodles, after the frying process, showed a lower proportion of carbohydrate, protein, fiber, and also total starch and digestible starch, but higher content of fat and resistant starch in the proximate analysis. The frying process was also considered to improve the texture, surface color, and sensory properties of instant noodle products, accompanied by better cooking quality, including shorter cooking time and lower cooking loss during the rehydration. The honeycomb-like, porous, and less uniformed structure, and also the higher levels of β-sheets and β-turns, and the lower proportion of α-helixes of protein structure from fried instant noodle was also observed. The *in vitro* digestibility of starch and protein were downregulated in the fried group (81.96% and 81.31, respectively, on average) compared with the non-fried group (97.58% and 88.78, respectively, on average). Thus, the frying process lowered the glycemic index and regulated protein secondary structure by inhibiting continuous digesting enzyme activity, generating starch-lipid complexes, and changing the levels of protein transformation. In conclusion, our findings will provide an innovative evaluation of the frying process on instant noodles and even other various starch-based prepared food products.

## Graphical Abstract

Construction of experimental methods and quality assessment system. 
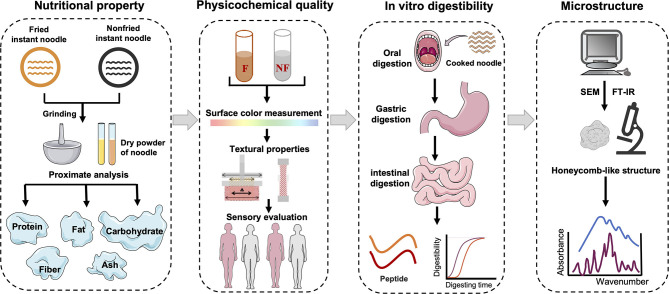

## Introduction

Noodle has been recognized as the second largest flour product with a slightly lower consumption than bread (1). In particular, instant noodle has become increasingly popular due to its excellent flavor and convenience in preparation, cooking, and delivery (2). Deep frying that involves a series of complex processes including Maillard and caramelization reaction, oxidation, polymerization, and hydrogenation promotes fried noodles to gain more popularity for their unique fried flavor, crispy texture, and golden appearance (3, 4). Recently, with consumers' increasing health concerns, high-oil content of fried food associated with a higher risk of developing chronic diseases, such as obesity, diabetes, and cardiovascular disorder, could be a potential factor influencing consumers' loyalty (5, 6), which make it necessary to systematically evaluate the effect of frying process on instant noodle and its nutritional attributes in human body.

Starch is the primary component of wheat flour, and the changes in its amount and structure give rise to the difference in nutrition, functionality, and palatability of instant noodle. Different dehydration treatments have been reported to influence the formation of starch-lipid complexes, thus altering their solubility (7). It is also noted that the formation of starch–lipid complexes during processing could affect the physicochemical qualities of starch, the texture, and digestibility of the final product (8). Besides, protein is another important factor that can contribute to the properties of instant noodles. For instance, some research indicated that instant noodles with higher protein content can exhibit firmer and more elastic texture (9), whereas others found that abnormal digestibility of cereal proteins could potentially reduce the bioavailability by forming the protein–fiber polymer and inhibiting the release of the peptide and amino acids, and thus negatively influencing the potentials of cereal proteins and the peptides derived from the hydrolysis of these premium proteins to lower the risks of obesity, type-2 diabetes, and cardiovascular diseases (10). To date, most of the research focus on optimizing the formula to improve the nutritional and sensory quality of fried instant noodles (11, 12). However, the mechanistic understanding of the effect of frying process on digestibility and structural characteristics of protein and starch remains limited, and their influence on the quality of final products also needs further investigation.

In this study, the measurement of proximate composition, optical properties, sensory evaluation, cooking qualities, and texture analysis were conducted to compare the nutritional value, physicochemical properties, and consumer acceptance of fried instant noodle (dehydrated by frying in oil) and non-fried instant noodle (removed the moisture by hot air drying). *In vitro* gastrointestinal digestion model was established, and the analysis of glycemic index, kinetic modeling, and proteolysis rate was also performed to explore the digestibility of starch and protein. Scanning electron microscopy (SEM) analysis and Fourier transform infrared spectroscopy (FT-IR) analysis were combined to obtain the microstructure of instant noodles during *in vitro* digestion.

## Materials and Methods

### Materials

Water used in all the experiments was obtained from a reverse osmosis water purification system. Trypsin (1,000–2,000 U/mg) and α-amylase (≥5,000 U/g) from the porcine pancreas were purchased from Sigma-Aldrich, Switzerland. Pepsin (≥250 U/mg) from porcine gastric mucosa, serine, and artificial saliva (pH = 6.8) were obtained from Solarbio, Beijing, CN. Pancreatin (≥4,000 U/g), o-phthalaldehyde, and bovine serum albumin were bought from Yuanye, Shanghai, CN. Ethanol and petroleum ether were purchased from Aladdin, Shanghai, CN. All the chemical reagents used were of analytical grade.

### Sample Collection and Preparation

All the samples used in this study were purchased from the online shopping channels of T-mall Supermarket. The selected instant noodle products, including fried instant noodle 1 (F1), fried instant noodle 2 (F2), fried instant noodle 3 (F3), non-fried instant noodle 1 (NF1), non-fried instant noodle 2 (NF2), non-fried instant noodle 3 (NF3), occupied the major market in their respective segments in China, and thus these 6 samples are the typical products for exploring the effect of frying process on nutritional property, physicochemical quality, and *in vitro* digestibility of commercial instant noodles. The detailed information and frying methods of instant noodle products can be found in Supplementary Material Table S1. After collection, the dry samples were obtained through being freeze-dried, ground, passed through a 100-mesh sieve, and stored at room temperature, whereas the wet samples were obtained after cooking in boiling water for 5 min.

### Proximate Composition

Moisture, protein, fat, and ash content of instant noodle samples were evaluated by constant weight method, Kjeldahl method, Soxhlet extraction method, and burning method, respectively, as the standard procedure from AOAC (13). Carbohydrate was calculated by subtracting the values of moisture, protein, fiber, and ash contents from 100. The fiber was measured with a Fiber Assay Kit (BC4285 Solarbio Co., Beijing, CN) according to the manufacturer's protocols.

### Physical Quality Assessments

#### Optics

The effect of frying on optic property of instant noodle products was determined by Colorimeter (YS6060, Sanen Co, Shenzhen, CN). Every noodle sample (5 g) was placed in the center of the glass plate for color measurement (L^*^, a^*^, and b^*^). The color of the noodle products was recorded with a precalibrated colorimeter at room temperature. The ΔE value was calculated according to the following equation, indicating the total color difference.


$$\begin{array}{l} △\text{E}=\sqrt{{\left({\text{L}}_{\text{s}}^{*}-{\text{L}}_{0}^{*}\right)}^{2}+{\left({\text{a}}_{\text{s}}^{*}-{\text{a}}_{0}^{*}\right)}^{2}+{\left({\text{b}}_{\text{s}}^{*}-{\text{b}}_{0}^{*}\right)}^{2}} \end{array}$$

where Ls^*^, as^*^, and bs^*^ are L^*^, a^*^, and b^*^ of the sample, and ${\text{L}}_{0}^{*}$, ${\text{a}}_{0}^{*}$, and ${\text{b}}_{0}^{*}$ are L^*^, a^*^, and b^*^ of the blank control.

#### Thermal Properties

The effect of frying process on the thermal properties of instant noodle products was determined according to Song et al. with differential scanning calorimetry (DSC, Metler Toledo, Schwerzenbach, Switzerland) (14). The noodles were freeze-dried, ground, and passed through a 100-mesh sieve. The powder (5 mg) and distilled water (15 mg) were mixed in a stainless-steel pan. DSC was performed from 20 to 200°C at a rate of 10 K/min, and then the heat flow and DSC curves were obtained.

#### Texture

The effect of frying on texture property of instant noodle products was evaluated according to Cao et al. using a TA-XT2i Texture Analyzer (Stable Micro System Ltd, United Kingdom) (11). A set of three cooked noodle samples was placed on the platform, measured with the HDP/PFS metal blade, and compressed until a compression ratio of 70% was reached with pretest speed, test speeds, posttest speeds of 4.0, 1.0, and 1.0 mm/s, respectively. Three replicates per sample were analyzed.

#### Cooking Quality

The effect of frying on cooking quality of instant noodle products was determined based on cooking time, cooking loss, and water adsorption. The cooking time was obtained when the opacity in the center of instant noodle products disappeared (15). The water adsorption was expressed as the percentage of increased weight compared with the raw noodle products (16), and the cooking loss was calculated by the amount of solid substance lost to cooking water (17).

### Sensory Evaluation

The effect of frying on sensory attributes (firmness, elasticity, slipperiness, chewiness, and overall acceptability) of cooked instant noodle products was analyzed as described by Sajad AhmadSofi with minor modifications (18). The healthy and taste-sensitive panelists were selected, excluding the patients with color blindness, rhinitis, stomatitis, and gastroenteritis. The sensory training was carried out, composed with basic sensory knowledge including common assessment methods and analysis, and also experimental skills, such as sample evaluation process and rules of scoring table. A total of 20 trained panelists comprising food scientists, research scholars, and students from the department of Tianjin Key Laboratory of Food Science and Health, Nankai University scored F1, F2, F3, NF1, NF2, and NF3 based on liking and disliking using a 9-point hedonic scale.

### *In vitro* Starch Digestibility and Estimated Glycemic Index

The effect of frying on starch digestibility of instant noodle products was determined according to the procedure of our previous study (19), modified from Englyst et al. (20). The starch digestibility was determined by measuring the content of glucose with the GOPOD kit (K-GLUC, Megazyme Bray, Co. Wicklow, Ireland). The content of rapidly digestible starch (RDS, ≤ 20 min), slowly digestible starch (SDS, 20–120 min), and resistant starch (RS, > 120 min) was also calculated. The degree of starch hydrolysis was fitted to the first-order rate equation:


$$\begin{array}{l} {\text{C}}_{\text{t}}=1-{\text{e}}^{-\text{kt}} \end{array}$$

where Ct is the amount of starch digested at time t (min), and k (min^−1^) is the first-order rate coefficient. The value of k can be calculated from the slope of a linear least-squares fit of a plot of ln (1 – Ct) against *t*.

The values were plotted on a graph and the area under the curve (AUC) was determined using GraphPad Prism 5 (GraphPad Software Inc., San Diego, CA, USA). The hydrolysis index (HI) of each sample was calculated by the following equation:


$$\begin{array}{l} \text{HI}=\frac{\text{AUC of sample}}{\text{AUC of reference food}} \end{array}$$

The expected glycemic index (eGI) was thus estimated using the following model:


$$\begin{array}{l} \text{eGI}=0.549\;\times \text{ HI}+39.71 \end{array}$$

### *In vitro* Protein Digestibility

The *in vitro* protein digestibility of instant noodle products was determined using a modified version described by Hur et al. (21). The phases of *in vitro* protein digestion model include mouth, stomach, and intestine, accompanied by the addition of artificial saliva (A7990, pH = 6.8, Solarbio Co., Ltd., Beijing, CN), and preprepared stimulated gastric, duodenal, and bile fluids, whose formulas are listed in Table 1.

**Table 1:** Constituents of the synthetic juices of the protein digestion model^a^.

	**Gastric juice**	**Duodenal juice**	**Bile juice**
Organic and inorganic components	1 g bovine serum albumin	1 g bovine serum albumin	1.8 g bovine serum albumin
		9 mL CaCl2·2H_2_O^b^	10 mL CaCl2·2H_2_ O
			30 g bile
Enzymes	2.5 g pepsin	9 g pancreatin	
	3 g mucin	1.5 g lipase	
pH	1.30 ± 0.02	8.1 ± 0.2	8.2 ± 0.2

a *After mixing all the ingredients (inorganic components, organic components, and enzymes), the volume was increased to 500 ml with distilled water*.

b *The concentration of CaCl_2_·2H_2_O reagent is 22.2 g/L*.

The degree of hydrolysis of the digested sample was analyzed using microplate OPA assay as described by Jadhav et al. (22). Appropriately 25 μL of diluted samples after centrifuging (10,000g, at 4°C for 20min) was mixed with 175 μL OPA reagent in the microtiter plate. The plate was incubated at 37°C for 2 min and the absorbance was measured at 340 nm. Standard was obtained using serine and degree of hydrolysis and was determined as follows:


$$\begin{array}{l} \text{Degree of hydrolysis }\left(\%\right)=\frac{{\text{h}}_{\text{sample}}}{{\text{h}}_{\text{total}}}\times 100\% \end{array}$$

$$\begin{array}{l} \begin{array}{l} {\text{h}}_{\text{s}}=\frac{Serine\;{\text{NH}}_{2}-\beta }{{\text{h}}_{\text{total}}}\times 100\% \end{array} \end{array}$$

$$\begin{array}{l} Serine\;{\text{NH}}_{2}=\;\frac{{\text{OD}}_{\text{sample}}-{\text{OD}}_{\text{blank}}}{{\text{OD}}_{\text{stand}}-{\text{OD}}_{\text{blank}}}\;\times \;0.9516\text{meqv/L }\\ \;\times \frac{0.1\times 100/g}{\text{X }\times \text{ P}} \end{array}$$

where h_sample_ is the concentration of free amino groups in the samples (mmol), h_total_ is the concentration of free amino groups per gram of completely hydrolyzed protein (8.83 mmol/g protein). X is the weight of the sample tested; P is the protein content of the sample tested; and α and β are expressed by the constants 1.00 and 0.40, respectively.

### Fourier Transform Infrared Spectroscopy Microscopy Analysis

Fourier transform infrared spectroscopy microscopy analysis of instant noodle products during *in vitro* digestion was carried out using the method of Wang et al. (23) with a Thermo Scientific Nicolet IS50 spectrometer (Thermo Fisher Scientific, Waltham, MA, U.S.A.). Noodle sheets from stimulated digesting phases of mouth, stomach, and intestine were freeze-dried, and a flat surface was obtained after treatment. Spectrums were collected 4 times with a resolution of 4 cm^−1^ in the wavenumber region between 4,000 and 400 cm^−1^. The spectral analysis referred to the methods described by Mehtap Fevzioglu with minor modifications (24). The secondary structure was processed by introducing second derivative techniques and Fourier-self deconvolution of the amide I region of the protein (1,600–1,700 cm^−1^) using OMNIC software (Thermo Electron Corp.).

### Scanning Electron Microscopy Analysis

The microstructure of the instant noodle products during *in vitro* digestion was determined by scanning electron microscope (SEM, S-3400N, Hitachi, Japan) with magnifications of × 500 and × 3,000 referred from Anju et al. (25). The instant noodle products were installed on the black ribbon, and the vacuum evaporator was used for sputtering gold plating. The ribbon with the sample was then placed in the SEM unit, and the images were captured at a different magnification at 15 kV accelerating voltage.

### Statistical Analysis

All the results are reported as the mean values and standard deviations (SDs). One-way analysis of variance (ANOVA) followed by Newman–Keul's multiple comparison test (*p* < 0.05) was conducted to determine the significant differences between mean values using GraphPad Prism 5 (GraphPad Software Inc., San Diego, CA, USA).

## Results

### Proximate Composition, Color, Thermal, Cooking Quality, and Texture Analysis

The proximate composition of instant noodle samples is shown in Table 2. The frying process increased the levels of fat content of F1, F2, and F3, but not in non-fried groups, whose fat was mainly derived from raw wheat flour. The carbohydrate content of F1, F2, and F3 was lower than NF1, NF2, and NF3. Compared with fried samples, non-fried instant noodle products contain higher protein and fiber content, especially NF2 has the highest protein (11.38 ± 0.04a) and fiber (1.14 ± 0.08a). The frying treatment did not have a significant effect on the ash content of these two types of instant noodle products, which might be related to different product formulations.

**Table 2:** Quality evaluation of fried and non-fried instant noodle (dry basis).

**Characteristic**	**Fried instant noodle**			**Non-fried instant noodle**		
	**F1**	**F2**	**F3**	**NF1**	**NF2**	**NF3**
**Proximate composition**						
Carbohydrate (%)	72.95 ± 0.11^c^	74.74 ± 0.10^c^	71.85 ± 0.84^c^	84.50 ± 0.19^a^	84.10 ± 0.36^b^	84.36 ± 0.12^a^
Fat (%)	17.49 ± 0.33^a^	12.86 ± 0.01^b^	17.05 ± 0.84^a^	1.95 ± 0.17^c^	2.51 ± 0.33^c^	2.72 ± 0.17^c^
Protein (%)	8.21 ± 0.18^e^	9.85 ± 0.03^b^	9.22 ± 0.04^c^	10.07 ± 0.02^b^	11.38 ± 0.04^a^	8.95 ± 0.01^d^
Ash (%)	1.22 ± 0.02^d^	2.14 ± 0.04^c^	1.33 ± 0.06^d^	2.37 ± 0.03^b^	0.74 ± 0.01^e^	3.12 ± 0.02^a^
Fiber (%)	0.12 ± 0.01^c^	0.41 ± 0.06^b^	0.55 ± 0.03^b^	1.05 ± 0.03^a^	1.14 ± 0.08^a^	0.87 ± 0.04^a^
**Texture**						
Hardness (N)	1025.53 ± 70.66^d^	1794.15 ± 22.15^b^	1200.57 ± 75.46^c^	2290.06 ± 23.33^a^	1801.65 ± 51.775^b^	2425.04 ± 7.51^a^
Springiness (N)	0.92 ± 0.02^c^	1.19 ± 0.19^b^	0.85 ± 0.01^d^	0.94 ± 0.02^b^	0.70 ± 0.05^e^	3.51 ± 0.19^ac^
Cohesiveness (N)	0.43 ± 0.02^c^	0.54 ± 0.02^b^	0.47 ± 0.01^c^	0.67 ± 0.01^a^	0.42 ± 0.01^c^	0.42 ± 0.01^c^
Chewiness (N)	3817.96 ± 18.53^a^	1269.37 ± 53.37^b^	1172.55 ± 10.59^b^	263.47 ± 33.67^e^	451.18 ± 8.90^d^	975.08 ± 69.53^c^
Gumminess (N)	978.08 ± 31.37^a^	937.25 ± 22.10^a^	530.24 ± 7.40^b^	1054.46 ± 4.95^a^	521.31 ± 68.66^b^	1087.37 ± 60.58^a^
Resilience (N)	0.28 ± 0.01^a^	0.20 ± 0.01^b^	0.18 ± 0.01^b^	0.13 ± 0.01^c^	0.17 ± 0.01^b^	0.17 ± 0.01^b^
**Cooking quality**						
Cooking time (s)	203 ± 2.0^d^	238 ± 2.5^c^	159 ± 1.0^e^	382 ± 1.5^a^	266 ± 0.5^b^	261 ± 1.0^b^
Cooking loss (%)	9.60 ± 0.37^b^	11.08 ± 0.30^b^	9.90 ± 0.05^b^	14.93 ± 0.62^a^	14.48 ± 0.55^a^	12.40 ± 0.21^b^
Water Absorption (%)	126.34 ± 3.42^e^	139.16 ± 0.13^d^	130.20 ± 0.49^e^	205.02 ± 1.75^a^	195.78 ± 2.10^b^	175.65 ± 0.11^c^
**Color**						
L*	87.08 ± 0.01^a^	84.92 ± 0.01^c^	84.45 ± 0.02^d^	85.85 ± 0.01^b^	84.15 ± 0.01^f^	84.30 ± 0.01^e^
a*	−0.36 ± 0.01^e^	−0.25 ± 0.02^d^	−0.17 ± 0.01^c^	0.73 ± 0.02^a^	0.64 ± 0.02^b^	0.70 ± 0.01^a^
b*	25.08 ± 0.03^c^	25.16 ± 0.02^b^	25.32 ± 0.01^a^	13.15 ± 0.01^f^	19.38 ± 0.01^d^	16.68 ± 0.01^e^
ΔE	14.15 ± 0.01^a^	13.39 ± 0.02^b^	13.40 ± 0.02^b^	5.41 ± 0.02^e^	7.76 ± 0.01^c^	5.63 ± 0.01^d^

*Results are reported in means ± standard deviations (n = 3). Results were analyzed using the Newman–Keuls test. Means with different letters in the same row are significantly different at p < 0.05*.

**Figure 1:**
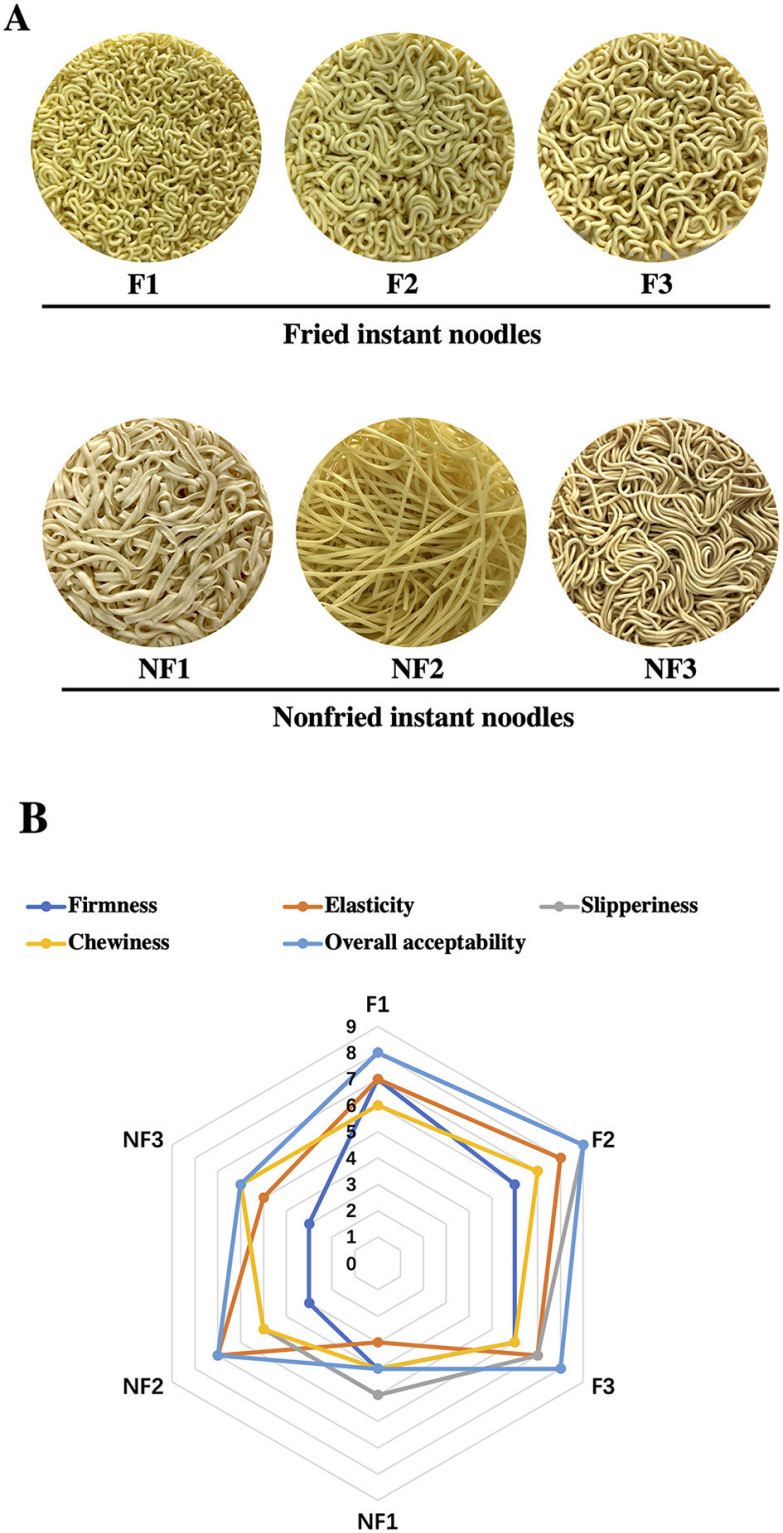
Preliminary evaluation of fried and non-fried instant noodle products. **(A)** appearance; **(B)** sensory evaluation.

Fried and non-fried noodles showed significant (*p* < 0.05) differences in color values, which are presented in Table 2 and Figure 1. Compared with non-fried noodle, F1, F2, and F3 showed a lower similarity to standard flour, with a significantly decreased yellowness (a^*^), an increased redness (b^*^), and an insignificant change in lightness (L^*^). In addition, the effect of the frying process on cooking qualities of instant noodle products can be seen in Table 2. The levels of cooking time, cooking loss, and water absorption capacity of NF1, NF2, and NF3 were significantly higher than those of F1, F2, and F3 (*p* < 0.01), probably due to the poor network development of non-fried instant noodle.

The DSC results of fried and non-fried noodle products showed typical gelatinization endotherms, and the thermal transition parameters including To (onset temperature), Tp (peak temperature), and ΔH (enthalpy change) can be found in Table 3 and Supplementary Figure S1. The onset temperature and peak temperature of F1, F2, and F3 were higher than that of NF1, NF2, and NF3, indicating that it is not easy to denature at lower temperatures for fried instant noodle products. The frying process also induced an increase in ΔH from 30.57 to 32.70 J/g and from 19.73 to 22.18 J/g, on average, inhibiting the degree of gelatinization due to the higher thermal stability of the fried instant noodle products.

**Table 3:** Thermal properties of fried and non-fried instant noodle.

**Characteristic**	**Fried instant noodle**			**Non-fried instant noodle**		
	**F1**	**F2**	**F3**	**NF1**	**NF2**	**NF3**
**Temperature (** **°** **C)**						
T_o1_	60.16 ± 0.57^a^	59.22 ± 1.07^a^	59.30 ± 0.29^a^	58.10 ± 0.29^a^	57.62 ± 0.39^a^	58.69 ± 0.31^a^
T_p1_	96.42 ± 1.44^a^	95.31 ± 0.33^a^	94.99 ± 0.58^a^	89.48 ± 1.44^b^	90.55 ± 0.21^b^	90.19 ± 0.42^b^
T_o2_ T_p2_	131.76 ± 0.29^a^ 158.02 ± 0.58^a^	131.44 ± 0.60^a^ 154.81 ± 0.94^a^	129.12 ± 0.58^a^ 154.13 ± 1.04^a^	122.18 ± 0.58^c^ 150.84 ± 0.31^b^	122.18 ± 1.15^c^ 149.98 ± 2.33^b^	126.54 ± 0.28^b^ 149.38 ± 0.57^b^
**Δ** **H (J/g)**						
Δ H_1_	32.24 ± 0.39^a^	33.36 ± 1.21^a^	32.53 ± 2.01^a^	29.60 ± 1.05^a^	31.99 ± 0.49^a^	30.12 ± 0.47^a^
Δ H_2_	23.08 ± 1.93^a^	21.36 ± 0.93^a^	22.09 ± 0.55^a^	18.95 ± 1.29^a^	20.96 ± 1.51^a^	19.28 ± 0.63^a^
Δ H_total_	55.32 ± 2.32^a^	54.72 ± 2.14^a^	54.62 ± 2.56^a^	48.55 ± 2.34^a^	52.95 ± 2.00^a^	49.40 ± 1.10^a^

*T_o_, onset temperature; T_p_, peak temperature; ΔH: enthalpy change of thermal transition. Results are reported in means ± standard deviations (n = 2). Results were analyzed using the Newman–Keuls test. Means with different letters in the same row are significantly different at p < 0.05*.

Furthermore, texture analysis has been widely used in the evaluation of food quality due to its high sensitivity and objectivity. According to the results in Table 2, the frying process had no significant intervention effect on the springiness, cohesiveness, and gumminess of the instant noodle, but could significantly downregulate the hardness and increase chewiness and resilience.

### Sensory Evaluation

The consumer acceptance of instant noodle products was evaluated based on sensory attributes, such as firmness, elasticity, slipperiness, chewiness, and overall acceptability appearance, using a 9-point hedonic scale (Figure 1B). Fried instant noodle products were more accepted, especially F2 had the highest sensory score for firmness (6), elasticity (8), slipperiness (9), chewiness (7), and overall acceptability (9), but all the non-fried samples showed a decline in all the sensory attributes and thus reduced overall acceptance.

### Starch Digestibility and Expected Glycemic Index

**Figure 2:**
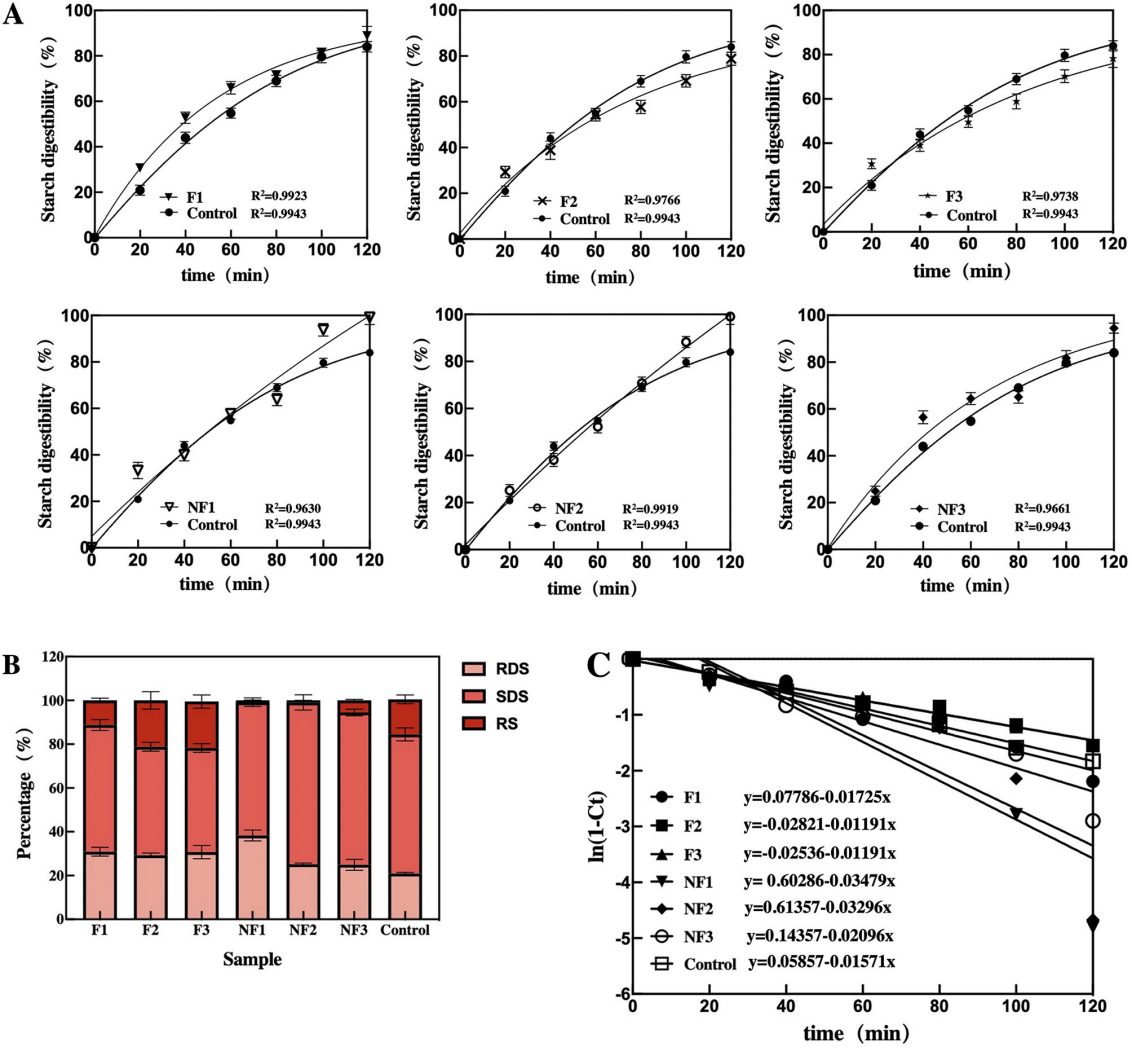
*In vitro* starch digestion of fried and non-fried instant noodle products. **(A)** Digestograms and the fit of first-order kinetics; **(B)** starch fractions **(C)** kinetic constants of instant noodle products.

The results of *in vitro* starch digestion are shown in Figure 2. All these starch digestograms of instant noodle products were characterized by a rapid digestion phase during the initial 40 min, followed by a slower digestion phase afterward (Figure 2A). There were no significant differences in the hydrolysis rate at an early stage, while the frying process lowered the degree of starch hydrolysis at a slower hydrolysis rate of instant noodle products afterward. The final digestion percentages at 2 h were 88.78%, 78.82, and 78.29% for fried instant noodle products (F1, F2, and F3 respectively), commonly lower than 99.18%, 99.06%, and 94.50% for NF1, NF2, and NF3 respective products without the frying process.

In addition, to gain a better mechanistic understanding of the enzymic digestion of starch from instant noodle products with frying methods, the digestion curves were fitted to the first-order kinetic equation (Table 4). *R*^2^ for fried and non-fried samples was above 0.9, ensuring that the enzymic digestion of the starches fitted well to first-order kinetics. Furthermore, the content of total starch (TS), digestible starch (DS), and RS can be seen in Table 4. The frying process lowered the levels of TS content and raised the proportions of RS. Based on the phases of *in vitro* hydrolysis, starch can be divided into RDS, SDS, and RS (Figure 2B). The RDS content of all these instant noodle products fluctuates between 24.91 and 38.30%, which was higher than the standard starch (20.94%).

**Table 4:** *In vitro* starch digestion and estimated glycemic index of fried and non-fried instant noodle.

**Characteristic**	**Fried instant noodle**			**Non-fried instant noodle**		
	**F1**	**F2**	**F3**	**NF1**	**NF2**	**NF3**
**Starch composition**						
TS (g/100g)	62.41 ± 0.26^d^	69.93 ± 0.70^c^	63.26 ± 0.11^d^	74.70 ± 0.14^a^	70.06 ± 0.10^c^	72.97 ± 0.04^b^
RS (g/100g)	11.22 ± 1.42^b^	21.18 ± 1.98^a^	21.71 ± 1.38^a^	0.82 ± 0.66^c^	0.94 ± 0.83^c^	5.50 ± 0.04^c^
DS (g/100 g)	51.19 ± 1.69^b^	48.74 ± 1.69^b^	41.56 ± 1.49^c^	73.89 ± 0.52^a^	69.11 ± 0.94^a^	67.47 ± 0.08^a^
**Nonlinear parameters**						
K × 10^−2^(min^−1^)	1.725 ± 0.183^e^	1.191 ± 0.086^d^	1.191 ± 0.075^d^	3.479 ± 0.901^a^	3.296 ± 0.844^b^	2.096 ± 0.353^c^
*R* ^2^	0.952	0.978	0.983	0.915	0.926	0.938
AUC Sample	6393 ± 22.84^b^	5766 ± 40.60^c^	5752 ± 29.61^c^	6804 ± 25.48^a^	6481 ± 19.26^b^	6794 ± 19.52^a^
HI	103.01 ± 0.73^b^	92.91 ± 1.28^c^	92.68 ± 0.93^c^	109.64 ± 0.81^a^	104.43 ± 0.60^b^	109.47 ± 0.61^a^
eGI	96.26 ± 0.40^b^	90.72 ± 0.70^c^	90.59 ± 0.51^c^	99.90 ± 0.44^a^	97.04 ± 0.33^b^	99.81 ± 0.34^a^

*TS, Total Starch; RS, Resistant Starch; DS, Digestible Starch; Values were expressed as the average of triplicates ± standard deviations. Results were analyzed using the Newman–Keuls test. Different letters within the same row are significantly different (p < 0.05)*.

The influence of the drying method on starch hydrolysis kinetics and the glycemic index of the instant noodle is shown in Table 4. Nonlinear parameters of the starch hydrolysis kinetics including kinetics constant (k), HI, and eGI can be found in Figure 1C and Table 4. The *k* value, representing the enzymatic hydrolysis rate or the susceptibility of starchy foods toward amylolysis (26), showed a significant difference in instant noodle products with and without the frying process. In this study, the values of k obtained from the slopes of the linear plots for F1, F2, and F3, ranged from 1.191 to 1.725 × 10^−2^ min^−1^, whereas it fluctuated from 2.096 to 3.479 × 10^−2^ min^−1^ for NF1, NF2, and NF3. Lower values of k indicated that the frying process probably can increase resistance to enzymatic starch hydrolysis, and obtain lower final starch digestibility (27). Besides, the frying process also reduced the eGI value (on average from 98.92 to 92.52).

### *In vitro* Protein Digestibility

**Figure 3:**
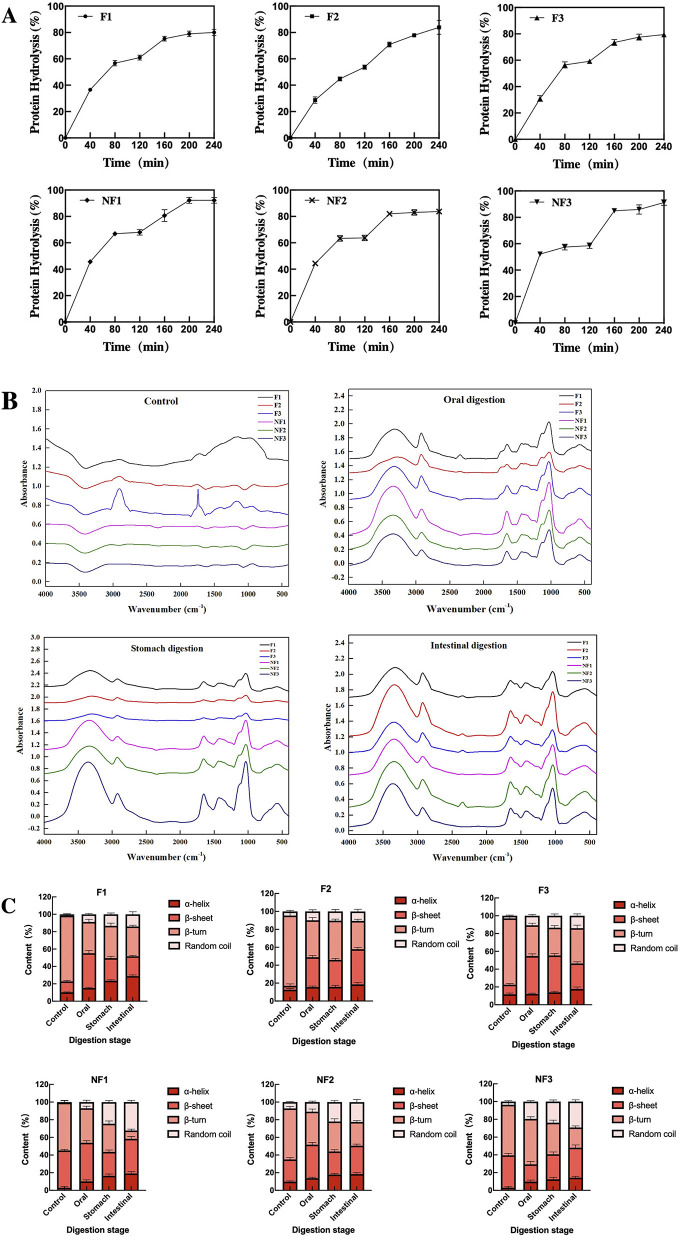
*In vitro* protein digestibility of fried and non-fried instant noodle products. **(A)** Protein hydrolysis rate of F1, F2, F3, NF1, NF2, and NF3, respectively; **(B)** FT-IR spectra during the phases of the untreated, oral, stomach, and intestine; **(C)** protein secondary structure changes during *in vitro* protein digestion.

The effect of frying on *in vitro* protein digestibility (IVPD) of instant noodle in oral, stomach, intestine digesting periods is shown in Figure 3A. During the *in vitro* gastric digestion, the frying process reduced the protein digestibility of F1, F2, and F3, but extended the digestive period probably due to the consistently higher activity of pepsin. In this case, non-fried instant noodle products almost reached the digestion peak at 80 min of digestion period, whereas the digestibility of fried instant noodles showed an upward trend throughout the digestion stage. Besides, during intestinal simulated digestion, the frying process also reduced the level of protein digestibility but did not significantly affect the tendency of protein hydrolysis. Although, IVPD of both these products reached a high level (over 80%) after gastrointestinal digestion, the lower level of protein digestibility of fried instant noodle products was observed at the end of digestion.

### FT-IR Spectroscopy and Protein Secondary Structure

Furthermore, FT-IR spectroscopy is a molecular vibrational spectroscopy that can monitor fine structural information about protein (28). The results for FT-IR of instant noodle products subjected to the frying treatment during the gastrointestinal digestion are shown in Figure 3B. There is a significant difference between the protein structure of fried and non-fried instant noodle. After the frying process, the spectrum peaks of undigested instant noodle products showed an irregular trend and remarkably increased the spectrum peak of instant noodle during gastric digestion at the wave number of 3,000–3,500 cm^−1^. The absorption peaks in the wavelength of 1,150 cm^−1^ and 850 cm^−1^ were also influenced by the frying process. The analysis of amide-I bands (1,600–1,700 cm^−1^) by second-derivative, Fourier self-deconvolution, and curve-fitting was also conducted to quantify the protein secondary structure of instant noodle (Figure 3C). Different special bands were observed in protein, including β-sheets (1,615–1,637 cm^−1^ and 1,682–1,700 cm^−1^), β-turns (1,664–1,681 cm^−1^), α-helixes (1,646–1,664 cm^−1^), and random coils (1,637–1,645 cm^−1^) (29). During the entire digestion process, the content of β-turns in fried instant noodle decreased significantly, but NF1, NF2, and NF3 did not show a similar trend without being fried. Furthermore, compared with non-fried instant noodle products, the frying process raised the levels of β-sheets, β-turns, and random coil, and lowered the percentage of α-helixes.

### Scanning Electron Microscope

**Figure 4:**
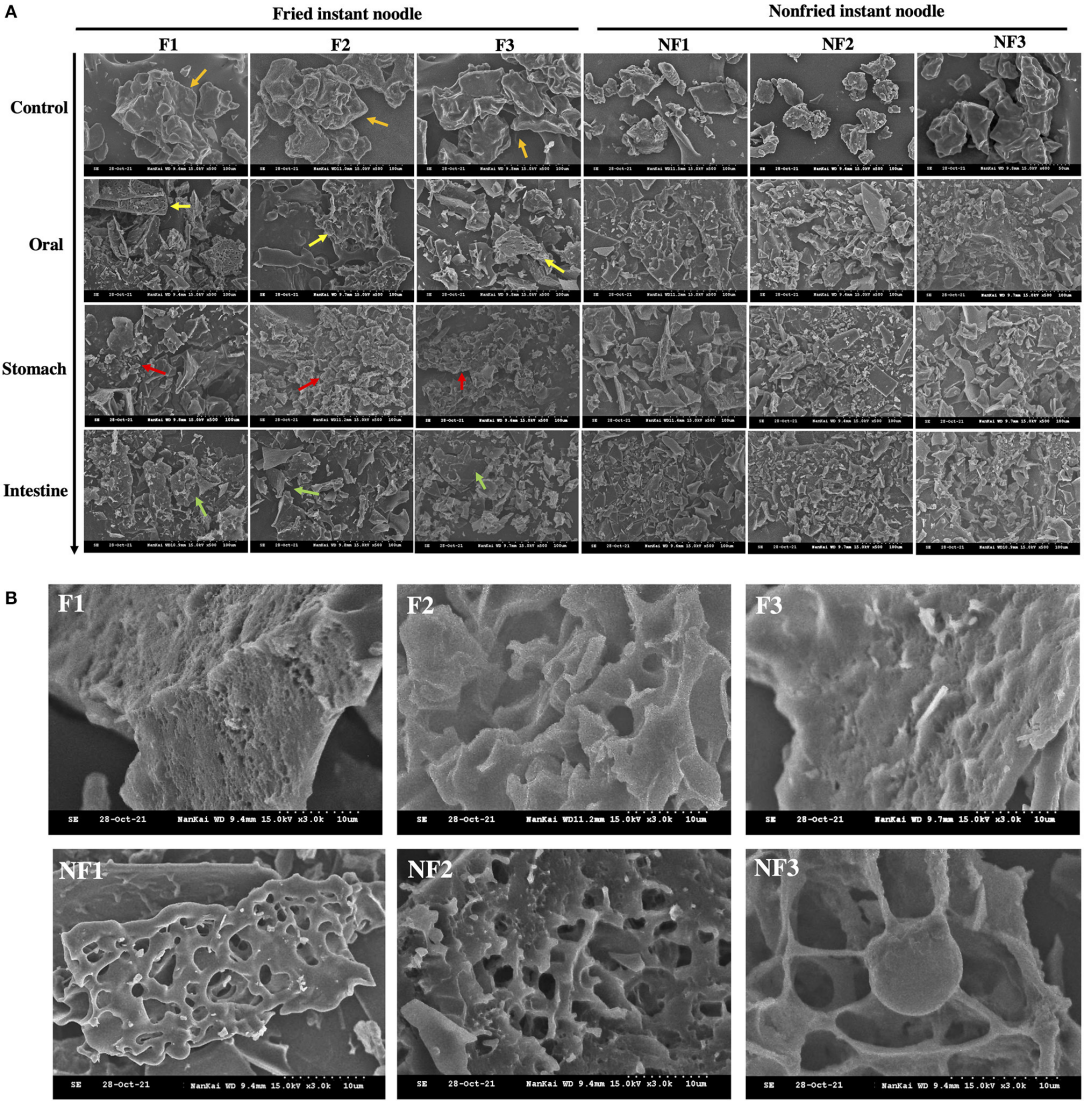
**(A)** Microstructure changes of fried and non-fried instant noodle products during simulated *in vitro* digestion (500×); **(B)** SEM image of instant noodle products at the final phase of digestion (3000×).

The effect of frying process on the internal microstructures of the instant noodle during *in vitro* digestion is shown in Figure 4A. It was observed that the native gluten particles had a composite structure with flat surfaces possibly due to the presence of starch–oil complexes (orange arrows), whereas the structure of non-fried instant noodle products has a higher degree of friability and disorder. Afterward, a visible honeycomb-like structure accompanied with numerous small pores inside (yellow arrows) was also formed. Next, many mushroom-like structures (red arrows) can be seen in the gastrointestinal digestion. This also remained incompletely digested particles (green arrows) for fried instant noodle products. In addition, the final microstructure of instant noodle during *in vitro* digestion was obtained at 3,000 × magnification (Figure 4B). It is obvious that the particulates inside fried instant noodle have been digested more thoroughly, compared with the integral structure in non-fried samples. The instant noodle products with frying process also presented a honeycomb-like appearance, whereas the surface structure of the non-fried instant noodle products had higher smoothness, uniformity, and continuity.

## Discussion

### Effect of Frying on Quality Evaluation of Instant Noodle

During the frying process, high temperature and oil mass transfer can affect the composition, structure, and physicochemical properties of the various ingredients in food (30). The lower content of carbohydrates in fried products indicates the flow of free sugar from food to oil during the frying process, which is in agreement with previous results (31). In terms of the protein, their spiral and folded structure might breakdown, and internal molecules might be degraded during frying. This also indicated that non-fried noodle is a better source of protein and fiber than fried noodle that can be used for the development of various functional food products (32). Although there is no significant difference in the ash content of fried and non-fried products, the similarly low ash content of these two kinds of instant noodle was noticed. This problem can be solved by supplementing the raw material and seasoning package with higher mineral content, suggesting an important direction for future research. Optical property is another one of the most efficient ways to evaluate the sensory quality, directly affecting consumer acceptance. More color changes of fried instant noodle may be caused by the Maillard reaction of the sugars and protein, and also due to the color polymer formed in the fat oxidation reaction during the frying process, so fried instant noodle presents a darker color with lower a^*^ and higher b^*^.

The frying process also decreased the levels of cooking time, water absorption, and cooking loss of instant noodle products. The lowered degree of water absorption capacity of fried instant noodle products mainly depended on the levels of starch gelatinization and decomposition of the molecular structure (33). Whereas non-fried instant noodle products have stronger water absorption capacity, which is likely due to the undamaged double helical structure and multiple spiral polymer secondary structure with higher water holding capacity. The decreased cooking loss for fried instant noodle might be attributed to the higher damaged starch of the flour blends and the formation of starch-lipid complexes, consistent with the previous study (12). It is also reported that the degree of cooking loss is related to solid leaching from the boiled noodles, which is considered an indicator of their overall cooking quality (14). In addition, for fried instant noodle, water can penetrate to the noodle inside from the pore structure formed during the frying process. Since the non-fried drying method cannot form a continuous and uniform pore structure, the water can only migrate from the surface layer of the noodle to the center with a lower degree of starch gelatinization and more difficulty for rehydration, leading to the shorter cooking time for fried instant noodle products.

The effect of frying process on the degree of gelatinization and decomposition of the starch complex can be seen from the higher To, Tp, and ΔH of fried instant noodle products. Previous studies have shown that the thermal transition temperatures of palm oil and wheat protein were around 40 and 60 °C, respectively (34). However, within the temperature scanning range of fried instant noodle products, there was no thermal transition peak, which meant that the free fatty acids and the wheat proteins had been decomposed. Once being fried, the starch gelatinization peak disappeared and a new peak can be found around 100 °C, indicating that the samples were gelatinized, and starch–lipid complexes were formed. Based on the calorimetry results, two distinctive peaks were obtained in the range of 89.48–96.42°C and 149.38–158.02 °C, respectively, presenting the formation of type I and type IIb complexes through melting and recrystallization, which showed a higher level of thermal stability compared with the non-fried samples (35). The increased peak temperature of instant noodle products, probably because of protein denaturation, would lead to structural extension, prevent the interaction between starch and other molecules, and thus interfere with the endothermic melting process of starch–lipid complexes. Furthermore, the melting enthalpy (ΔH) of the starch–lipid complex could reflect the amount of complex and the degree of order within the complex (36). The higher level of ΔH for fried instant noodle products indicated that the frying process provides more energy to activate the interaction between starch and lipid molecules, resulting in the formation of more starch–lipid complexes with higher stability and order.

The texture profile analysis is one of the most effective methods to evaluate the edible quality of cooked noodle products. This is probably because the starch–lipid complexes formed during the frying process could disrupt the regular gluten network structure in the dough, thus weakening the hardness of fried samples. The association between texture and protein and fiber content of fried instant noodle was observed, probably because the monomeric molecules inside the protein had a significant positive relationship with the hardness of cooked instant noodle (37), whereas higher fiber content has been confirmed to increase the strength of the dough by interacting with the gluten system, leading to increased hardness of non-fried cooked noodles (38). Moreover, chewiness and resilience are affected by starch properties (total amount, molecular structure, and distribution within the tuber), starch swelling pressure, and gelatinization properties (39, 40). These might be the reasons why fried noodle showed a downward trend in the performance of hardness, and an upward trend in the performance of chewiness and resilience, compared with non-fried instant noodle.

In terms of sensory evaluation, fried instant noodle products have more acceptable performance than non-fried products. Some of the possible reasons are as follows: Dough of fried instant noodle containing starch and protein is usually cooked at a high temperature above 130°C at which Maillard and caramelization reaction will occur, producing more flavors and pigments, which contributes to the preliminary evaluation of instant noodle products (41). Many of these end-products are fat-soluble, easily dissolving in the fried products after the frying process, and so the aroma might be more lasting and softer (42). The oil itself could also produce the fragrant substance, such as 2, 4-decadienal after thermal decomposition, which produces a unique flavor for instant noodle products (43). Additionally, the presence of more fat for instant noodle after the frying process could have a positive effect on the smoothness, uniformity, and continuity of the noodle surface, thus improving the overall sensory performance of instant noodles (44).

### Effect of Frying on Starch Digestibility of Instant Noodle

The performance of starch for instant noodle products, including the rate and degree of hydrolysis, components, and eGI, represents the capacity of energy supply, sugar regulation, and intestinal absorbance. First, the frying process downregulated the starch hydrolysis. This might be attributed to the formation of the starch–lipid complex that is considered RS type 5 (45). This type of complex obtained after the frying process showed higher RS, which stays in line with the results shown in Table 3. Besides, the frying process significantly decreased the TS content of instant noodle, possibly due to the degradation of the starch molecules during deep-frying, as observed in other studies (46). The lower levels of TS content brought by the frying process would result in changes in the thermodynamic, crystalline, and pasting properties of the starch (47), which is another one of the reasons leading to lower starch digestibility for instant noodle products. Apart from this, compared with standard starch, the higher RDS content of instant noodle products may be due to the dilution effect on starch gelatinization and the enhancement of starch–protein interaction caused by the frying process (48). Frying treatment could reduce the SDS content and increase the RS content. Although higher RS content of fried instant noodle products cannot be absorbed in the small intestine, it could be fermented and metabolized by intestinal microorganisms in the large intestine and promote human health by regulating the lipid metabolism, cholesterol synthesis, fatty acid oxidation, and other physiological processes in a variety of ways (49), so the higher RS content can been recognized as one of the nutritional attributes of instant noodle products, compared with other wheat-made food.

According to the WHO, foods with a GI value less than 55 are recognized as low-GI foods and those with over 70 are high-GI foods (50). All these high-GI foods, especially those with GI values of over 90, can enter the gastrointestinal tract with a rapid digestible rate, thus raising the blood sugar rapidly. Interestingly, our results showed a significant reduction in GI when the instant noodle products were fried, compared to non-fried products. This GI variation might be negatively correlated with RS, and this contributes to the nutritional properties for fried noodle products because the diet with high-GI food has been considered as one the of risk factors leading to Type 2 diabetes (51, 52), whereas non-fried instant noodle is still the priority on the diet for marathon runners and patients with hypoglycemia when the body urgently needs energy, and this can quickly supply energy and raise the blood sugar.

### Effect of Frying on *in vitro* Protein Digestibility of Instant Noodle

The frying process can change the protein digestibility, internal structure, and external interaction for instant noodle products by regulating the degree and rate of protein hydrolysis and the proportion of secondary conformation. During the *in vitro* protein digestion, the lower digesting rate of fried instant noodle at an early stage might be since the frying process allows the lipid to enter the noodle products, which can quickly remove the moisture and create small pores carrying the air, thus inhibiting the rapid contact between pepsin and the noodle surface. The lower digesting terminal point of fried instant noodle might be related to protein oxidative modification caused by the pores carrying the air, just as demonstrated before. This would easily lead to the intermolecular cross-linking and protein aggregation, and weaken the combination of digestive enzymes and proteins, thus reducing protein digestibility. Moreover, the protein hydrolysis could release peptide chain and hydrophilic molecules, most of which are hydrophilic and likely to prevent further contact of the protein with trypsin in the presence of oil after the frying process. To sum up, although the frying process reduced the protein digestibility, IVPD of both these products reached a high level after gastrointestinal digestion. Proteins with high IVPD are usually regarded as high-quality proteins because proteolysis facilitates the release of amino acids from the protein backbone, which means they can be better digested and absorbed (53).

Besides, based on the irregular peaks of fried instant noodle products in the results of FT-IR, the wave number of 3,000–3,500 cm^−1^ represents the amide-A and free hydroxyl groups, indicating the interaction of protein molecules with water (54). This increased spectrum peak change of fried instant noodle products is probably because of some structural modifications caused by the frying process. While the absorption peaks in the wavelength of 1,150 and 850 cm^−1^ represent C–O stretching and C–H deformation vibration, respectively, (55), the degradation and new interactions of protein molecules might be responsible for these different peaks of the spectrum. As for the protein secondary structure, the lowered content of β-turns of fried instant noodle products indicated that protein continuously unfolded, allowing the protein to aggregate to the molecular scale. An increment in β-sheets content of fried instant noodle products was also observed, which was a sign of molecular aggregation (56). The higher levels of β-turns, random coil, and lower percentages of α-helixes of fried instant noodle could interrupt the structure of hydrogen bonds, affect the dehydration of gluten, and prevent direct contact between protein molecules and enzymes, thus negatively affecting the final protein digestibility.

### Effect of Frying Process on Microstructure of Instant Noodle

The SEM was applied to detect the structural changes, formation of lipid–starch complex, and the levels of hydrolysis during *in vitro* stimulated digestion. The frying process promoted the generation of pores in the interior of the noodles, and this might be attributed to the formation of larger vapor pressure when water evaporates at the early stage of the frying process, which destroyed the internal structure and produced numerous pores. The honeycomb-like structure accompanied with numerous small pores inside observed after oral digestion was formed probably because of the glassy transition and the uneven shrinkage during the frying process (57). These pores created by the frying process allow digestive enzymes to enter inside, so that the noodles can be digested quickly afterward, while the existence of complete gluten structure in non-fried instant noodle made it difficult to digest with an outside-in approach. This is one of the reasons why fried instant noodle products have a higher digestion rate at the beginning. While during the gastrointestinal digestion, structural modifications caused by the frying process prevented continuous digestion, mushroom-like structures were formed that attributed to the incompletely digested particles for fried instant noodle products, thus reducing the final hydrolysis rate.

## Conclusion

The effect of frying process on the nutritional property, physicochemical quality, and *in vitro* digestibility of instant noodle products was investigated in this study. Results clearly revealed that frying reduced the proportion of carbohydrate, protein, fiber, ad also TS and DS, but increased the content of fat and RS. The results also suggested that the frying process decreased the *in vitro* digestibility of starch and protein, and the levels of expected glycemic index because frying was confirmed to promote the interaction between starch and lipid molecules, resulting in the formation of type I and type IIb complexes through melting and recrystallization. We have also observed that the frying process could form a honeycomb-like structure, prevent continuous contact with digesting enzymes, and thus intervene in the *in vitro* digestion of instant noodle products through generating starch–lipid complexes, changing the degree of gelatinization, and transforming the protein structure. Accordingly, the frying process conferred more acceptable texture, color, and sensory attributes to the final consumer products, accompanied with better cooking quality including shorter term of cooking time and lower cook loss during rehydration, resulting from the properties of protein and starch (total amount, molecular structure, and digestibility), presence of the lipid, and higher levels of fat-soluble color polymer. Taken together, this study will contribute to the innovative evaluation of the frying process on instant noodles and other various starch-based prepared food products, and further research is necessary to address the mechanisms underlying the involved intermolecular interaction and provide further development of fried products based on optimized frying process.

## Data Availability Statement

The original contributions presented in the study are included in the article/Supplementary Material, further inquiries can be directed to the corresponding author/s.

## Author Contributions

JW and AL designed this study, performed the experiments, and prepared the manuscript. JH, BZ, and JL coordinated the laboratory work and prepared the tables and figures. SW and YZ aided in the analysis and interpretation of the data. All authors agree to be accountable for the content of the work and approve the manuscript.

## Funding

This work was supported by grants from the National Natural Science Foundation of China (No. 32030083).

## Conflict of Interest

The authors declare that the research was conducted in the absence of any commercial or financial relationships that could be construed as a potential conflict of interest.

## Publisher's Note

All claims expressed in this article are solely those of the authors and do not necessarily represent those of their affiliated organizations, or those of the publisher, the editors and the reviewers. Any product that may be evaluated in this article, or claim that may be made by its manufacturer, is not guaranteed or endorsed by the publisher.

## Abbreviations

SEM: scanning electron microscopy
FT-IR: Fourier transform infrared spectroscopy
RDS: rapidly digestible starch
SDS: slowly digestible starch
RS: resistant starch area under curve (AUC)
HI: hydrolysis index
eGI: expected glycemic index
OPA: O-phthalaldehyde
TPA: texture profile analysis
TS: total starch
DS: digestible starch
IVPD: *in vitro* protein digestibility.
