# Supplementary material for: Effect of Frying Process on Nutritional Property, Physicochemical Quality, and in vitro Digestibility of Commercial Instant Noodles
Source: Front Nutr. 2022 Feb 17;9:823432. doi: 10.3389/fnut.2022.823432 (PMC8891372; doi:10.3389/fnut.2022.823432)
Supplement: Supplementary file 1 [file Table_1.DOC]

***Supplementary Material***

1. **Detailed information of fried and nonfried noodles**

Table S1. Detailed information of fried and nonfried noodles

| Grouping | Company | Brand | Product name | Ingredient  (excluding seasoning package) | Purchase means | Purchase site |
| --- | --- | --- | --- | --- | --- | --- |
| Fried instant noodle 1（F1） | Tingyi (Cayman Islands) Holding Co., Ltd. | Mr. Kon | Braised beef noodles | Wheat flour, palm oil, starch, acetate starch, salt, gluten, sodium pyrophosphate, xanthan gum, hydroxymethyl cellulose, vitamin E, Na_2_CO_3_, K_2_CO_3_, (NaPO_3_)_6_, NaH_2_PO_4_, Na_5_P_3_O_10_ | Online | https://www.tmall.com |
| Fried instant noodle 2（F2） | Tingyi (Cayman Islands) Holding Co., Ltd. | Master Tang | Barbecued pork bone noodles | Wheat flour, palm oil, starch, starch acetate, salt, gluten, soy protein, xanthan gum, sodium alginate, konjac flour, whole egg powder, gardenia yellow, riboflavin, vitamin E, Na_2_CO_3_, K_2_CO_3_, (NaPO_3_)_6_, NaH_2_PO_4_, Na_5_P_3_O_10_ |
| Fried instant noodle 3（F3） | Tingyi (Cayman Islands) Holding Co., Ltd. | Master Tang | Wolfberry and flower maw chicken noodles | Wheat flour, palm oil, starch, acetate starch, salt, gluten, compound thickening stabilizer, sodium carboxymethyl cellulose, sodium pyrophosphate, soy protein, xanthan gum, sodium alginate, konjac flour, whole egg powder, gardenia yellow, riboflavin, vitamin E, Na_2_CO_3_, K_2_CO_3_, (NaPO_3_)_6_, NaH_2_PO_4_, Na_5_P_3_O_10_, |
| Nonfried instant noodle 1（NF1） | Tingyi (Cayman Islands) Holding Co., Ltd. | Mr. Kon | Beef fresh noodles | Wheat flour, starch acetate, salt, egg powder, sunflower seed oil, soluble soybean polysaccharide, lactose, phospholipids, maltodextrin, guar gum, distarch phosphate, Na_5_P_3_O_10_, vitamin E, vitamin C |
| Nonfried instant noodle 2（NF2） | Jinmailang Mianpin Co., Ltd. | Mr. Fan | Braised beef noodles | Wheat flour, starch acetate, salt, gluten, phospholipids, polyglycerin fatty acid ester, guar gum, gardenia yellow, propylene glycol alginate, Na_2_CO_3_, NaH_2_PO_4_, (NaPO_3_)_6_ |
| Nonfried instant noodle 3（NF3） | Nissin Foods Holdings Co., Ltd. | King of ramen | Black garlic oil pork bone noodles | Wheat flour, starch, vegetable oil, salt, carotene, gum arabic, sodium glutamate, gum arabic, vitamin E, Na_2_CO_3_, CaCO_3_, (NaPO_3_)_6_, Na_5_P_3_O_10_, |

1. **Frying process of fried instant noodle products**

Noodle powder ingredients were dissolved, mixed, compressed through the manufacturing model, shaped, and dehydrated by the high temperature frying. Generally, the oil temperature of onset point, midpoint, and end point was 125~130 ℃, 140~145 ℃, 158~163 ℃, respectively, and this whole process usually lasted for 105~115 s. The final fried instant noodle products were obtained based on the above process.

1.
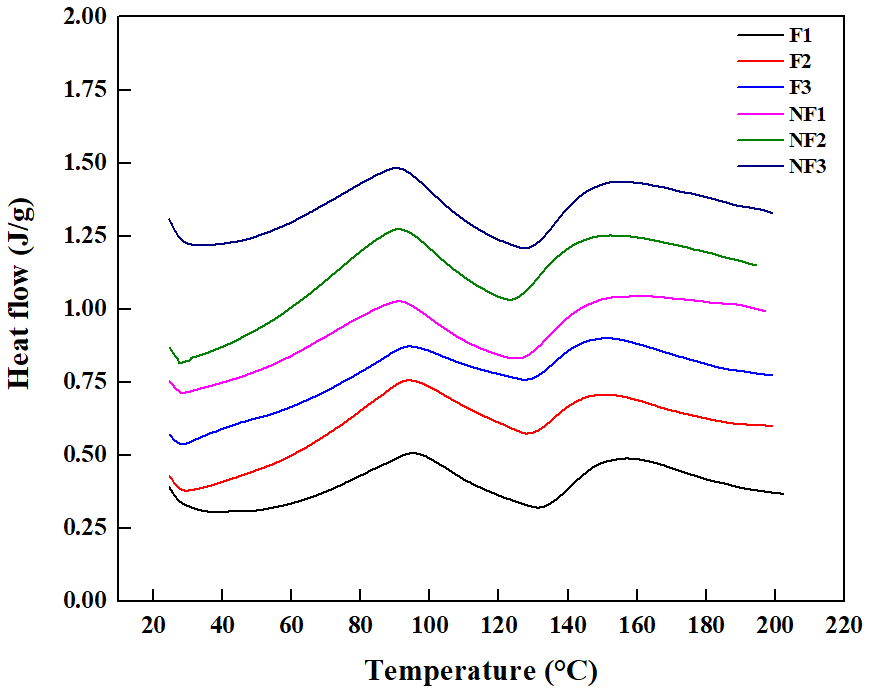
**Thermal Properties of fried and nonfried instant noodle products**

Figure S1. DSC thermograms of fried and non-fried instant noodle products
